# Emerging roles of the solute carrier family in pancreatic cancer

**DOI:** 10.1002/ctm2.356

**Published:** 2021-03-24

**Authors:** Zijian Wu, Jin Xu, Chen Liang, Qingcai Meng, Jie Hua, Wei Wang, Bo Zhang, Jiang Liu, Xianjun Yu, Si Shi

**Affiliations:** ^1^ Department of Pancreatic Surgery Fudan University Shanghai Cancer Center Shanghai China; ^2^ Department of Oncology Shanghai Medical College Fudan University Shanghai China; ^3^ Shanghai Pancreatic Cancer Institute Shanghai China; ^4^ Pancreatic Cancer Institute Fudan University Shanghai China

**Keywords:** pancreatic cancer, solute carrier, tumor biology

## Abstract

Pancreatic cancer is a gastrointestinal tumor with a high mortality rate, and advances in surgical procedures have only resulted in limited improvements in the prognosis of patients. Solute carriers (SLCs), which rank second among membrane transport proteins in terms of abundance, regulate cellular functions, including tumor biology. An increasing number of studies focusing on the role of SLCs in tumor biology have indicated their relationship with pancreatic cancer. The mechanism of SLC transporters in tumorigenesis has been explored to identify more effective therapies and improve survival outcomes. These transporters are significant biomarkers for pancreatic cancer, the functions of which include mainly proliferative signaling, cell death, angiogenesis, tumor invasion and metastasis, energy metabolism, chemotherapy sensitivity and other functions in tumor biology. In this review, we summarize the different roles of SLCs and explain their potential applications in pancreatic cancer treatment.

## Graphical abstract

Pancreatic cancer is a rare but highly fatal disease with poor prognosis. Some solute carriers (SLCs) have been identified to participate in several steps of pancreatic tumorigenesis, which would be a hot spot of future research and serve as new therapeutic strategies.

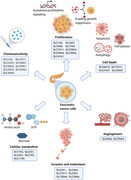

**Abbreviations**
5‐FU: 5‐fluorouracil
AIF: apoptosis‐inducing factor
ASCT2: alanine–serine–cysteine transporter 2
CREB: cAMP response element‐binding protein
EMT: epithelial–mesenchymal transition
GLUT1: glucose transporter 1
HGNC: HUGO Gene Nomenclature Committee
LAT1: L‐type amino acid transporter 1
MgtE: magnesium (Mg^2+^) transporter E
MMPs: matrix metalloproteases
mTOR: mammalian target of rapamycin
NBC3: bicarbonate (Na^+^/HCO_3_^–^) cotransporter 3
NRP‐1: neuropilin‐1
PCD: programmed cell death
PDTC: pyrrolidine dithiocarbamate
Pfam: protein family
PTGR2: prostaglandin reductase 2
ROS: reactive oxygen species
RREB1: Ras responsive element‐binding protein 1
SGLT1: sodium‐dependent glucose transporter 1
SLC: solute carrier
STAT3: signal transducer and activator of transcription 3
TPEN: *N*,*N*,*N'*,*N'*‐tetrakis (2‐pyridylmethyl) ethylenediamine
VEGF: vascular endothelial growth factor
VNUT: vesicular nucleotide transporter
ZIP: Zrt‐IRF‐like proteins
ZnT: zinc transporter protein

## 1 BACKGROUND

Although pancreatic cancer has a low incidence of ∼2.5%, it is a highly fatal malignancy with a 5‐year overall survival rate of ∼8%, making it the seventh leading cause of cancer‐related death worldwide; it accounts for 458,918 (2.5%) new cases and 432,242 (4.5%) new deaths annually according to GLOBOCAN 2018.^1^, ^2^, ^3^ However, because of the location of the pancreas, deep in the abdomen, and the limited number of typical symptoms and lack of early and accurate detection methods, many patients are diagnosed with advanced or metastatic stages; consequently, only 15–20% of patients with pancreatic cancer are eligible for surgical resection at diagnosis.^4^ In recent decades, some progress has been achieved in the diagnosis, perioperative management, and radiotherapy and systemic therapy approaches for advanced diseases, but these advances have had limited effects on overall survival outcomes.^5^ Therefore, the molecular mechanism of pancreatic cancer tumorigenesis is a key point in pancreatic oncology research. Studies on solute carriers (SLCs) in cancer pharmacology and drug discovery have become a hotspot in recent years, particularly due to their roles as chemotherapeutic drug targets or mediators of drug disposition. Since studies by Li et al. revealed the significant role of SLC39A4 in pancreatic cancer tumor progression, our interest in research on SLCs in pancreatic cancer has grown.^6^

## 2 INTRODUCTION TO SLC AND THEIR RELATIONSHIP WITH CANCER

The SLC superfamily encodes the second largest group of membrane transport proteins after G protein‐coupled receptors, comprising 65 families with over 400 SLC transporters reported in humans to date.^7^, ^8^, ^9^ Most of these proteins are located in the membranes of cells and organelles, and their main functions are to maintain cellular homeostasis by facilitating the exchange of a wide variety of soluble molecular substrates across lipid membranes. SLC transporters rely on electrochemical gradients or ion gradients to transport a diverse array of substrates, including glucose, amino acids, vitamins, nucleotides, inorganic ions, organic ions, neurotransmitters, and drugs.^8^, ^10^, ^11^ The nomenclature for SLCs is based on the combination and overlap of two databases: the HUGO Gene Nomenclature Committee (HGNC) (http://www.genenames.org/) and the SLC tables (http://www.bioparadigms.org/slc/intro.htm). The HGNC provides each human gene with a certain approved symbol that also curates it into large families based on its substrates, structure, mechanism, and other characteristics.^7^, ^12^ SLCs usually have the gene symbol name that represents the superfamily and the original protein name, for example, SLC39 and Zrt‐IRF‐like proteins (ZIPs). SLCs with over 20% protein sequence identity are classified into the same family.^13^ Concerning the species, two multiorganism classifications have been developed: the transporter classification (TC) system (http://www.tcdb.org/) based on functional and phylogenetic information of transporter proteins and the protein family (Pfam) system (http://pfam.xfam.org/) based on protein sequence alignment using Markov models. SLCs are divided by phylogenetic analyses into four groups denoted as α, β, δ, γ and simultaneously clustered into five Pfam clans by sequence similarity, namely, major facilitator superfamily (MFS), amino acid/polyamine/organocation (APS), cation proton antiporter/anion transporter (CPA/AT), drug/metabolite transporter (DMT), and others without clans.^14^ Additionally, the transporters have also been divided into two types according to the intracellular and extracellular concentrations of their substrates: passive (facilitated) transporters mediate the passage of substrates down their electrochemical gradients without using energy and active transporters (symporters and antiporters) create gradients by utilizing energy. Members of the SLC superfamily play crucial roles in human physiology through process such as the intake of amino acids in the intestine for cell growth, absorption of glucose for cellular energy metabolism, transport of metal ions to serve as essential cofactors for enzymes, and uptake of water‐soluble vitamins for vital processes or reabsorption of neurotransmitters into presynaptic neurons.^8^ Given the significant roles of SLCs in regulating normal cellular functions, dysregulation of certain SLC proteins is associated with various diseases, including diabetes mellitus, hypertension, chronic kidney disease, dermatosis, cancer, and many others.^13^

**FIGURE 1:**
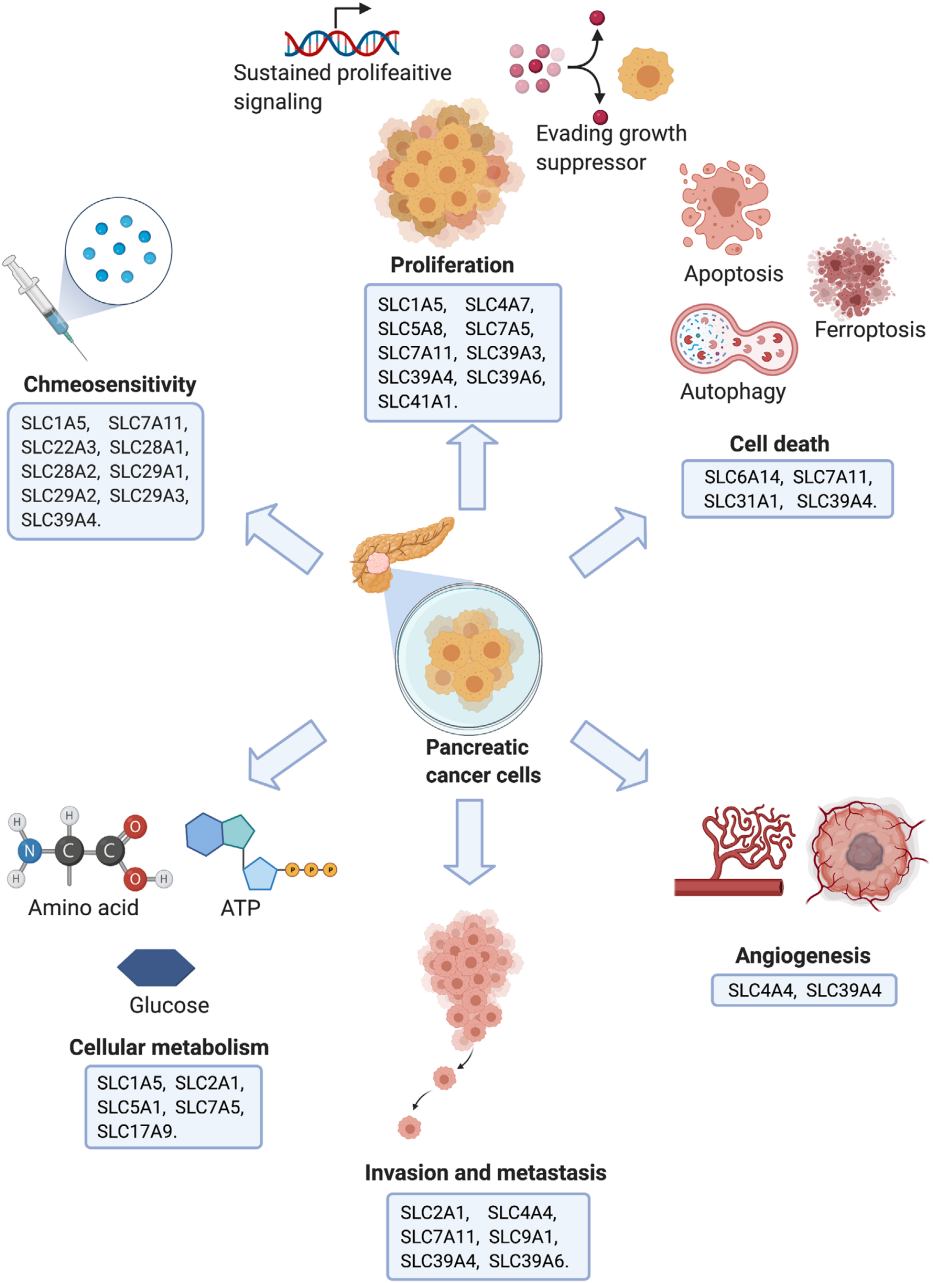
Functions of solute carriers (SLCs) in pancreatic tumor biology

According to the hallmarks of cancer proposed by Hanahan and Weinberg, tumorigenesis is a multistep process that gradually transforms normal cells into highly malignant derivatives.^15^, ^16^ Previous studies have reported that the SLC superfamily participates in various steps of tumorigenesis, including proliferation, apoptosis, invasion and metastasis, chemotherapy resistance, and other processes related to cancer.^10^ The overexpression or suppression of SLCs may provide new strategies for determining the diagnosis, treatment, or prognosis. Some tumors must transport nutrients into cells to fuel their growth and survival, and thus the inhibition or activation of SLC transporter function may be a possible therapeutic strategy. In addition, some SLC transporters have the ability to deliver certain drugs to cancer cells, suggesting that they can serve as new targets for improving chemotherapy sensitivity and overcoming drug resistance. The next few sections aim to summarize examples of SLCs that have been reported to play a role in one or more hallmarks of pancreatic tumor biology and describe their functions (Figure 1 and Table 1).

**TABLE 1:** List of solute carriers (SLCs) playing different roles in pancreatic cancer

**Gene name**	**Aliases**	**Protein name(s)**	**Substrates**	**Transport type**	**Promote/suppress**	**Mechanism**	**Other phenotypes**	**References**
1. Proliferative signaling								
SLC1A5	System ASC	ASCT2	L‐Ala, L‐Ser, L‐Cys, L‐Thr, L‐Gln, L‐Asn	Na^+^/amino acid cotransporter/exchanger	Promote	HIF‐2α, mTOR	5	^17^, ^19^
SLC4A7	NBC2, NBC3, SLC4A6	NBCn1	Na^+^, HCO_3_^–^	Na^+^/HCO_3_^–^ cotransporter	Promote	Ras, TGFβ/EMT	NA	^20^
SLC7A5	System L	LAT1	Large neutral L‐amino acids, T3, T4, L‐DOPA, BCH	Amino acid exchanger	Promote	mTOR, c‐Myc	5	^21^ ^23^, ^24^, ^25^
SLC7A11	System X_c_^−^	xCT	Cys (anionic form), L‐glutamate	Amino acid exchanger	Promote	PI3K/Akt, STAT	2, 4, 6	^27^
SLC39A4	NA	ZIP4	Zn^2+^	NA	Promote	IL‐6/STAT3	2, 3, 4	^6^, ^31^, ^32^, ^33^, ^34^
SLC39A6	NA	ZIP6/LIV‐1	Zn^2+^	NA	Promote	STAT3/EMT	NA	^35^
SLC5A8	AIT	SMCT1	Short chain fatty acids	Na^+^/Cl^–^ cotransporter	Suppress	NA	NA	^41^, ^42^
SLC39A3	NA	ZIP3	Zn^2+^	NA	Suppress	REEB1	NA	^43^, ^44^, ^45^, ^46^
SLC41A1	NA	MgtE	Mg^2+^	Channel‐like	Suppress	Akt/mTOR	NA	^50^
2. Cell death								
SLC6A14	ATB^0,+^	NA	Neutral, cationic amino acids	Na^+^/Cl^–^‐dependent	Suppress	mTOR	1	^61^
SLC7A11	System X_c_^−^	xCT	Cystine, L‐glutamate	Amino acid exchanger	Suppress	ROS	1, 4, 6	^53^, ^54^
SLC31A1	CTR1	hCtr1, Ctr1, COPT1	Cu^2+^, cisplatin	K^+^‐dependent	Suppress	ROS	NA	^60^
SLC39A4	NA	ZIP4	Zn^2+^	NA	Controversy	Caspase‐3, 7, 8, 9	1, 3,4	^56^, ^57^, ^58^
3. Sustained angiogenesis								
SL4A4	NBC, NBC1	NBCe1	Na^+^, HCO_3_^–^	Na^+^/HCO_3_^–^ cotransporter	Suppress	NA	4	^63^
SLC39A4	NA	ZIP4	Zn^2+^	NA	Promote	VEGF	1, 2, 4	^32^, ^64^
4. Invasion and metastasis								
SLC2A1	NA	GLUT1	Glucose, galactose, mannose, glucosamine	Facilitated transporter	Promote	AMPK/FOXO3A/PUMA, MMP‐2	5	^69^, ^70^, ^71^
SLC4A4	NBC, NBC1	NBCe1	Na^+^, HCO_3_^–^	Na^+^/HCO_3_^–^ cotransporter	Promote	NA	3	^63^
SLC7A11	System X_c_^−^	xCT	Cys (anionic form), L‐glutamate	Amino acid exchanger	Promote	PI3K/Akt	1, 2, 6	^27^
SLC9A1	APNH	NHE1	Na^+^, Li^+^, H^+^, NH_4_^+^	Na^+^/H^+^ exchanger	Promote	EGFR	7	^73^, ^74^, ^75^
SLC39A4	NA	ZIP4	Zn^2+^	NA	Promote	ZO‐1/claudin‐1	1, 2, 3	^76^
SLC39A6	NA	ZIP6/LIV‐1	Zn^2+^	NA	Promote	STAT3/EMT	1	^35^
5. Cellular metabolism								
SLC1A5	System ASC	ASCT2	L‐Ala, L‐Ser, L‐Cys, L‐Thr, L‐Gln, L‐Asn	Na^+^/amino acid cotransporter/exchanger	Promote	HIF‐2α	1, 6	^17^
SLC2A1	NA	GLUT1	Glucose, galactose, mannose, glucosamine	Facilitated transporter	Promote	hnRNPA2/B1/PKM2	4	^68^, ^69^, ^71^, ^80^, ^81^, ^82^
SLC5A1	NA	SGLT1	Glucose, galactose	Na^+^/glucose cotransporter	Promote	AMPK/mTOR	NA	^83^, ^84^
SLC7A5	System L	LAT1	Large neutral L‐amino acids, T3, T4, L‐DOPA, BCH	Amino acid exchanger	Promote	c‐Myc	1	^25^, ^85^, ^86^
SLC17A9	NA	VNUT	Purine nucleotides	Cl^–^‐dependent	Promote	NA	NA	^92^
6. Chemotherapy sensitivity								
SLC1A5	System ASC	ASCT2	L‐Ala, L‐Ser, L‐Cys, L‐Thr, L‐Gln, L‐Asn	Na^+^/amino acid cotransporter/exchanger	Promote	HIF‐2α	1, 5	^17^
SLC7A11	System X_c_^−^	xCT	Cys (anionic form), L‐glutamate	Amino acid exchanger	Promote	ROS	1, 2, 4	^98^, ^99^
SLC22A3	EMT (uptake‐2 system)	OCT2	Organic cations	Facilitated transporter	Promote	NA	NA	^101^
SLC22A7								^128^
SLC28A1	NA	CNT1	Nucleosides	Na^+^/nucleoside cotransporter	Suppress	NA	NA	^105^
SLC28A2	NA	CNT2	Nucleosides	Na^+^/nucleoside cotransporter	Suppress	NA	NA	^105^
SLC29A1	NA	ENT1	Nucleosides	Facilitated transporter	Controversy	NA	NA	^102^, ^114^
SLC29A2	NA	ENT2	Nucleosides	Facilitated transporter	Suppress	NA	NA	^115^
SLC29A3	NA	ENT3	Nucleosides	NA	Suppress	NA	NA	^116^
SLC39A4	NA	ZIP4	Zn^2+^	NA	Promote	STAT3/ZEB1/JNK/ENT1	1, 2, 3, 4	^117^

Abbreviations: Ala, alanine; Asn, asparagine; Cys, cystine; ENT1, equilibrative nucleoside transporter 1; Gln, glutamine; MgtE, magnesium (Mg2+) transporter E; NA, not available; Ser, serine; SGLT1, sodium‐dependent glucose transporter 1; TGFβ, transforming growth factor β; Thr, threonine; VNUT, vesicular nucleotide transporter.

### 2.1 Proliferation

The most fundamental characteristic of cancer cells is their unlimited proliferation ability. Normal cells carefully regulate the generation and release of growth‐promoting signals, ensuring cellular homeostasis and maintaining the normal structure and function of the tissue. However, cancer cells control their own fate by deregulating these signals and disrupting normal checkpoints, thereby allowing the cells to proliferate continuously.^16^ Alternatively, cancer cells themselves produce growth factor ligands and bind to cognate receptors to promote proliferation in an autocrine manner. In addition, these cells also upregulate receptor expression, resulting in a hyperresponse to growth factor ligands.^16^

SLC1A5 encodes the alanine–serine–cysteine transporter 2 (ASCT2) protein and is a Na^+^‐dependent neutral amino acid cotransporter that mainly functions to transport glutamine and other neutral amino acids into mitochondria.^17^, ^18^ Kaira et al. evaluated 97 surgically resected samples from patients with pancreatic cancer and concluded that the expression of ASCT2 in the pancreatic cancer group was significantly higher than that in the benign pancreatic lesion group.^19^ A recent study also showed that oncogenic growth was dramatically suppressed by the knockdown of SLC1A5 and that overexpression of this gene restored cell growth, which indicated a critical role for SLC1A5 in pancreatic tumor growth.^17^

SLC4A7, also known as electroneutral Na^+^/HCO_3_^–^ cotransporter 3 (NBC3), regulates the transport of sodium and bicarbonate ions at a 1:1 ratio and plays a critical role in regulating the intracellular pH. In a recent study published in *Nature*, researchers found that knockout of SLC4A7 in Ras mutant pancreatic ductal adenocarcinoma (PDAC) cells induces micropinocytosis to support tumor growth.^20^

**FIGURE 2:**
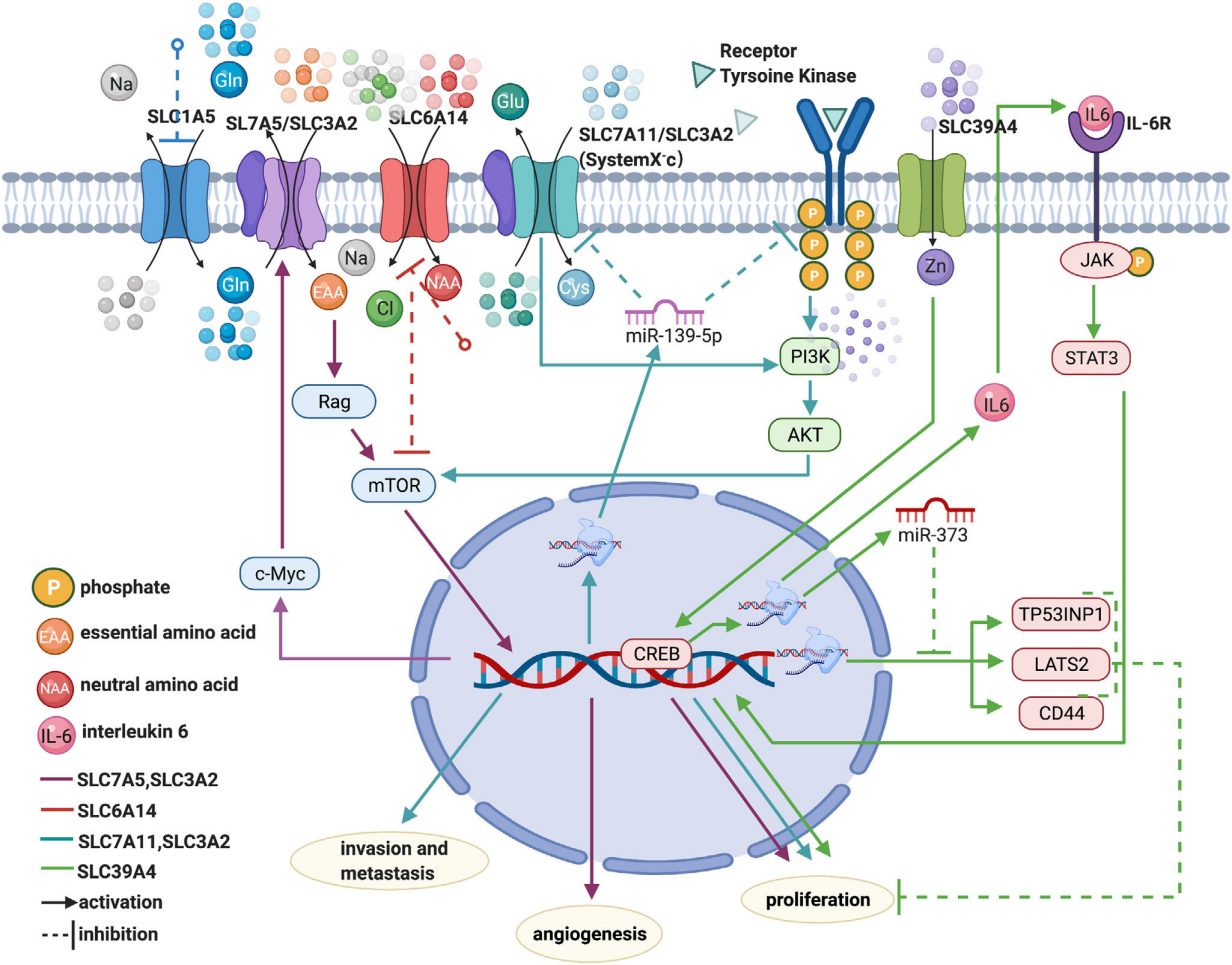
Solute carriers (SLCs) associated with PDAC cell proliferation, angiogenesis, invasion, and metastasis. (A) Glutamine is transported into tumor cells by SLC1A5 and can be used as an efflux substrate by the SLC7A5/SLC3A2 complex to mediate the uptake of essential amino acids (EAAs), finally activating the mTOR pathway to promote cell proliferation and angiogenesis.^21^, ^23^, ^24^ Overexpression of c‐Myc induces the expression of SLC7A5, which further activates mTOR signaling.^25^ (B) The SLC7A11/SLC3A2 complex (system X_c_^−^) regulates the uptake of cystine against the efflux of L‐glutamate, which further activates the PI3K/Akt signaling pathway to promote cell proliferation.^26^ The proliferation of tumor cells is also inhibited by miR‐139‐5p expression via the PI3K/AKT signaling pathway.^27^ (C) Overexpression of SLC39A4 upregulates the transcription of IL‐6 through CREB, which activates STAT3 to increase cell proliferation and tumor progression.^34^ SLC39A4 induces the transcription of miR‐373, which inhibits the transcription of TP53INP1, LATS2, and CD44 to promote tumor growth.^34^ (D) Inhibition of SLC6A14 decreases tumor cell proliferation by suppressing the mTOR pathway^61^

SLC7A5, which encodes the light chain of the L‐type amino acid transporter 1 (LAT1) protein, is a Na^+^‐independent amino acid transporter that regulates the influx of L‐leucine and the efflux of L‐glutamine when it interacts with SLC3A2.^21^, ^22^ Another clinicopathological study by Kaira et al. showed that high expression of CD147 was associated with the expression of SLC7A5 and SLC1A5, which correlated with proliferation, angiogenesis, and mammalian target of rapamycin (mTOR) signaling in pancreatic cancer cells.^23^ Fuchs and Bode hypothesized that LAT1 provides essential amino acids for tumor growth and proliferation and is overexpressed in cancer tissues via the mTOR signaling pathway, whereas SLC1A5 drives the function of SLC7A5 through glutamine delivery.^24^ As shown in a study by Nicklin et al., L‐glutamine activates mTOR signaling to mediate cell growth and proliferation, a process that is regulated by the transporters SLC1A5 and SLC7A5/SLC3A2.^21^ The results of a study by Hayashi et al. also revealed the biological significance of c‐Myc in pancreatic cancer growth, indicating that overexpression of c‐Myc increases SLC7A5 activity by binding to a canonical sequence in the promoter (Figure 2).^25^

SLC7A11 encodes the light chain subunit xCT, which forms a complex with the cystine/glutamate antiporter system X_c_^−^ and the heavy chain subunit SLC3A2. The main function of the X_c_^−^ system is to promote glutathione biosynthesis through the uptake of cystine and the concomitant release of glutamate.^26^ Zhu et al. indicated that the proliferation of pancreatic cancer cells is promoted by SLC7A11 overexpression and inhibited by miR‐139‐5p expression via the PI3K/AKT signaling pathway.^27^

SLC30, which encodes zinc transporter proteins (ZnTs) and SLC39, which encodes ZIPs, are members of two zinc transporter families in human cells with opposite functions.^28^ ZnTs mediate zinc flow from the cytoplasm to the extracellular environment or absorb zinc from the cytoplasm in the intracellular compartment to reduce the cytosolic zinc concentration, while ZIPs mediate the absorption of zinc from the extracellular environment and transport into the cytoplasm or induce the release of zinc from intracellular organelles (including the endoplasmic reticulum, mitochondria, and Golgi) into the cytoplasm to increase the cytosolic zinc concentration.^29^, ^30^

Regarding SLC39A4 (ZIP4), Li et al. first reported in 2007 that the ZIP4 mRNA is overexpressed 5.5‐fold in pancreatic cancer tissue specimens compared with normal tissues, and ZIP4 overexpression was also confirmed to increase cell proliferation by 101% in pancreatic cancer tissue.^6^ Then, in 2009, the authors further deciphered the function of SLC39A4 by silencing it in a mouse pancreatic cancer model and found that cell proliferation, migration, and invasion were all suppressed and that survival outcomes were significantly improved.^31^ However, the detailed mechanism by which SLC39A4 regulates pancreatic cancer growth is not completely clear. Zhang et al. explained a potential mechanism in their study: SLC39A4 mediates pancreatic cancer cell growth by upregulating the expression of neuropilin‐1 (NRP‐1), vascular endothelial growth factor (VEGF), and matrix metalloproteases (MMPs) in cell lines and xenografts.^32^ In addition, Zhang et al. revealed another mechanism: overexpression of SLC39A4 increases the transcription of interleukin 6 (IL‐6) through cAMP response element‐binding protein (CREB), which activates signal transducer and activator of transcription 3 (STAT3) and increases the expression of cyclin D1 to increase cell proliferation and tumor progression in pancreatic cancer.^33^ Furthermore, they identified a novel mechanism in 2013 in which ZIP4 transcriptionally induced miR‐373 expression in pancreatic cancer to promote tumor growth by activating CREB, which formed a novel signaling pathway.^34^

Another zinc transporter, SLC39A6, which encodes the ZIP6 protein (also known as LIV‐1), is a downstream target of STAT3 that is crucial for the regulation of the epithelial to mesenchymal transition (EMT). Unno et al. reported higher expression of the LIV‐1 mRNA in both pancreatic cancer cell lines and cancer tissues than in normal cell lines and tissues.^35^ Moreover, downregulation of SLC39A6 inhibits pancreatic cancer cell proliferation in vitro and reduces tumor growth and metastasis in vivo, which indicates the aggressive role of SLC39A6 in pancreatic cancer.

In addition to sustaining proliferative signaling, cancer cells also have the capability to circumvent tumor suppressor genes that limit tumor cell growth and proliferation. SLC5A8, also known as sodium‐coupled monocarboxylate transporter 1 (SMCT1), is a plasma membrane cotransporter for monocarboxylates such as lactate, butyrate, pyruvate, acetate, propionate, nicotinate, and β‐hydroxybutyrate.^36^, ^37^, ^38^, ^39^ Among its substrates, butyrate, propionate and pyruvate are histone deacetylase (HDAC) inhibitors that function as tumor suppressors by inducing apoptosis.^40^ Park et al. reported that aberrant promoter DNA methylation induces the silencing of SLC5A8 in pancreatic cancer, which may contribute to the carcinogenesis and progression of pancreatic tumors.^41^ Similarly, Helm et al. assessed the effect of SLC5A8 expression on the survival outcomes of patients with pancreatic cancer and found that low expression and nuclear translocation of SLC5A8 were associated with a poor prognosis.^42^

Regarding SLC39A3, Costello et al. reported that the physiological zinc level exerts cytotoxic effects on pancreatic cancer cells and that the downregulation of Ras responsive element‐binding protein 1 (RREB1) represses the expression of SLC39A3, resulting in a decrease in zinc levels, which alleviates these cytotoxic effects and promotes the proliferation of pancreatic cancer cells.^43^, ^44^ An in vivo study by Takagishi et al. indicated that ZIP3 expression is regulated by RREB1 in normal ductal/acinar epithelial cells.^45^ Similarly, Franklin et al. found that concurrent downregulation of SLC39A3 and its positive regulator RREB1 decreases zinc uptake and increases cell proliferation in pancreatic cancer cells.^46^

SLC41A1, the homolog of the prokaryotic magnesium (Mg^2+^) transporter E (Mgt E) family, was the first discovered as a sodium‐dependent eukaryotic protein that mediates the efflux of Mg^2+^ in human cells.^47^, ^48^, ^49^ Xie et al. reported that overexpression of SLC41A1 suppressed the growth of human PDAC cell lines by inducing the expression of Bax while suppressing the expression of Bcl‐2.^50^ In addition, overexpression of SLC41A1 increased the efflux of cellular Mg^2+^ and suppressed the activation of Akt/mTOR signaling, which regulates Bcl‐2 upstream.^50^

### 2.2 Cell death

Programmed cell death (PCD) describes the death of a cell via apoptosis, which serves as a natural barrier for cancer cell development.^51^, ^52^ PCD is a regulated process that usually confers advantages during an organism's life cycle. The second important characteristic of cancer cells is their ability to evolve to limit or circumvent PCD.^16^ Most cells evade apoptosis through a loss of function mutation of the tumor suppressor gene TP53, upregulation of the expression of the antiapoptotic genes Bcl‐2 or Bcl‐x_L_ and survival signals (IGF1/2), downregulation of proapoptotic genes (Bax, Bim, and PUMA), or the inhibition of ligand‐induced cell death.^16^

In addition to cell proliferation, SLC7A11 (xCT) also participates in PCD in pancreatic cancer. Chang et al. reported that the inhibition of prostaglandin reductase 2 (PTGR2) induces reactive oxygen species (ROS)‐dependent apoptosis by suppressing the expression of SLC7A11 (xCT).^53^ Similarly, in 2020, a study by Badgley et al. revealed the mechanism of ferroptosis (a form of cell death caused by lipid ROS) in pancreatic cancer in mice.^54^ They found that knockdown of SLC7A11 (xCT) in engineered mice induced tumor cell ferroptosis and repressed pancreatic cancer tumor growth, indicating that pancreatic cancer cells rely on cysteine import to evade ferroptosis, which may be a promising anticancer therapeutic approach for pancreatic cancer.^54^

According to previous studies, zinc is a crucial trace element that regulates cell proliferation, apoptosis, cellular metabolism, and gene expression in cancer.^55^ In a report by Cui et al., SLC39A4 conferred apoptosis resistance in pancreatic cancer cells through the caspase‐9/caspase‐7/PARP cascade.^56^ Donadelli et al. also found that use of the zinc chelator *N*,*N*,*N'*,*N'*‐tetrakis (2‐pyridylmethyl) ethylenediamine (TPEN) to deplete cellular zinc levels induced pancreatic cancer cell apoptosis by regulating the expression of mitochondria‐related genes in the Bcl family (Bcl‐X, Bcl‐2, Bcl‐W, and Bcl‐XL), and this effect was reversed by zinc addition.^57^ The authors confirmed that TPEN induced cell death by activating the caspase‐3 and caspase‐8 pathways.^57^ However, they also reported that the application of the ionophore compound pyrrolidine dithiocarbamate (PDTC) increased zinc levels and inhibited the growth of pancreatic cancer cells by inducing ROS‐dependent cell apoptosis.^58^ Their experiments also showed that zinc‐dependent ROS production induced mitochondrial damage through the nuclear translocation of mitochondrial apoptosis‐inducing factor (AIF), which results in cell apoptosis instead of caspase pathway activation.^58^ Therefore, the role of zinc in preventing pancreatic cancer cell apoptosis requires further study.

Another potent mechanism by which SLC mediates resistance to cell death/apoptosis is via cancer cell autophagy. SLC31A1, also known as CTR1, is a cell membrane transporter that regulates the intracellular copper concentration.^59^ Yu et al. found that copper intake is important for the progression of pancreatic cancer and that the knockdown of SLC31A1 blocked copper absorption to inhibit pancreatic cancer progression by increasing ROS‐induced autophagy, which also contributed to resistance to cell apoptosis.^60^

**FIGURE 3:**
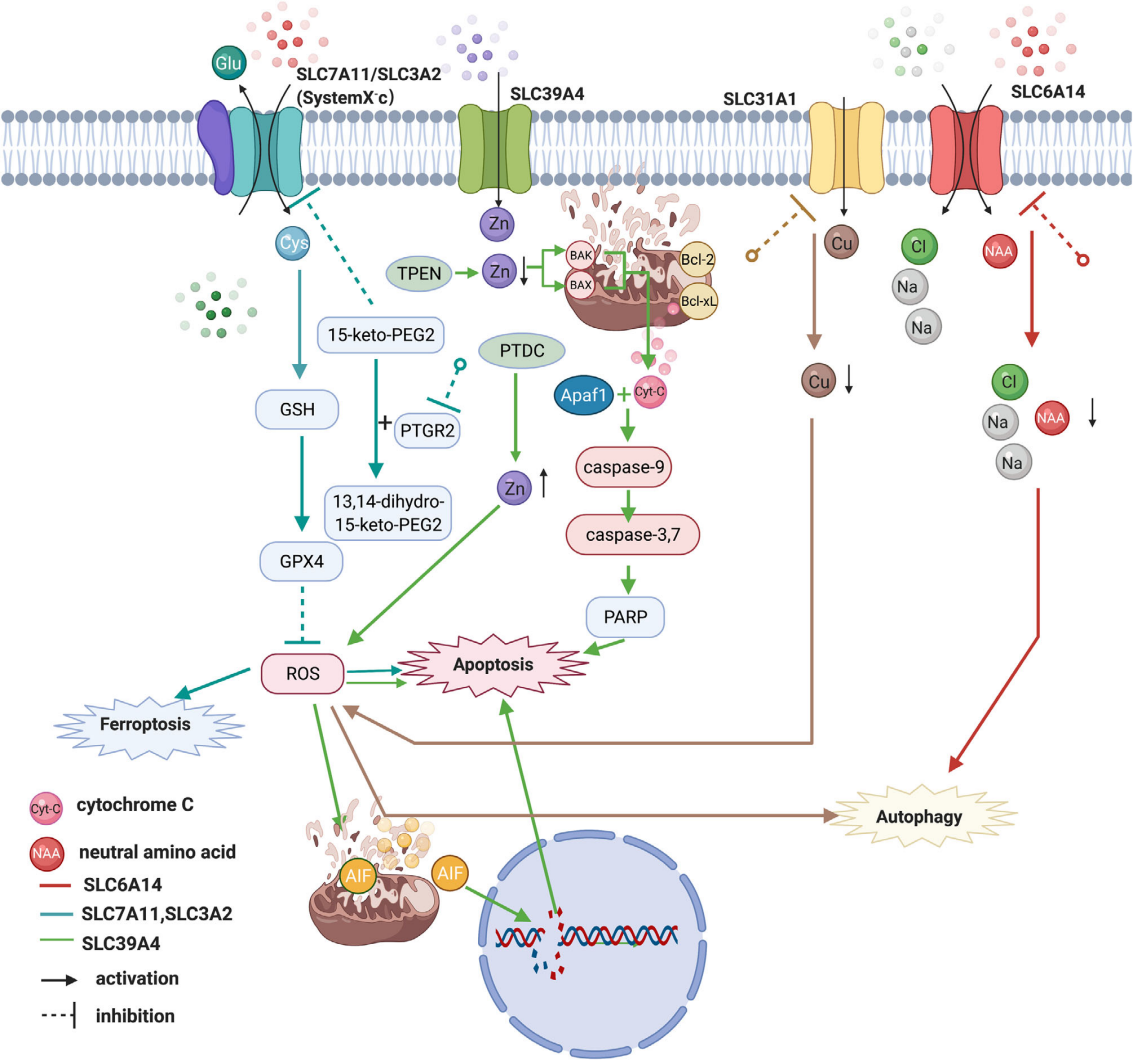
Solute carrier (SLC) expression is associated with cell death. (A) Inhibition of PTGR2 suppresses the expression of the SLC7A11/SLC3A2 complex (system X_c_^−^), resulting in the activation of ROS‐dependent apoptosis.^53^ The suppression of SLC7A11 (xCT) also induces ferroptosis via lipid ROS production.^54^ (B) Cyt‐C, which is released as a result of mitochondrial damage caused by Bax and Bak, binds to Apaf1 to activate the caspase‐9/caspase‐3/caspase‐7/PARP pathway and induce cell apoptosis.^56^ Overexpression of SLC39A4 increases zinc levels and represses the expression of Bax and Bak, subsequently conferring cell apoptosis resistance.^56^ Conversely, the use of TPEN to reduce zinc levels induces apoptosis by inhibiting the antiapoptotic genes Bcl‐2, Bcl‐W, and Bcl‐XL.^57^ Paradoxically, the use of pyrrolidine dithiocarbamate (PDTC) to increase zinc levels also induces apoptosis through ROS‐induced mitochondrial damage mediated apoptosis‐inducing factor (AIF), resulting in cell apoptosis instead of caspase pathway activation.^58^ (C) The inhibition of SLC31A1 to block copper absorption leads to the suppression of pancreatic cancer progression by increasing ROS‐induced autophagy, which also promotes the resistance to cell apoptosis.^60^ (E) Blockade of SLC6A14 induces tumor cell autophagy^61^

SLC6A14, also known as amino acid transporter B^0,+^ (ATB^0,+^), mediates the uptake of a broad range of neutral and cationic amino acids in a Na^+^/Cl^−^‐dependent manner.^61^ As shown in a study by Coothankandaswamy et al., pharmacological and genetic inhibition of SLC6A14 decreases the proliferation of pancreatic cancer cells both in vitro and in vivo, and blockade of this gene induces tumor cell autophagy and suppresses the mTOR signaling pathway (Figures 2 and 3).^61^

### 2.3 Angiogenesis

In addition to the traits mentioned above, tumor cells must also absorb oxygen and nutrients and release carbon dioxide and waste, which could occur via tumor angiogenesis.^16^ The process of angiogenesis in normal cells is transiently activated in certain situations, such as wound healing or endometrial repair; however, in tumor cells, angiogenesis is always activated. The essential cytokine mediating this process is VEGF, a signaling protein secreted by tumor cells to stimulate the creation of new blood vessels, which is associated with tumor metastasis and a poor prognosis.^32^

SLC4A4, which encodes the protein NBCe1, is a sodium bicarbonate transporter that was identified as a target of microRNA‐223.^62^ Zhang et al. reported that overexpression of human circular RNA 001587 upregulates the expression of SLC4A4 to inhibit angiogenesis in pancreatic cancer by binding to microRNA‐223.^63^

In addition to its function in cell proliferation, SLC39A4 is reported to upregulate the expression of VEGFs in pancreatic cancer by upregulating its receptor NRP‐1, revealing an autocrine signaling pathway involving the ZIP4–VEGF–NRP‐1 axis.^32^, ^64^

However, limited studies have been conducted on SLCs and their involvement in the angiogenesis of pancreatic cancer, necessitating further studies.

### 2.4 Invasion and metastasis

After tumors are formed at the original site, malignant cells can leave the primary site via different mechanisms and travel (metastasize) to distant tissues or organs with environments suitable for their growth. This process contributes to the death of most patients with cancer.^65^, ^66^, ^67^ Several SLCs have been reported to regulate the invasion and metastasis of pancreatic cancer.

SLC2A1‐encoding glucose transporter 1 (GLUT1) mediates glucose uptake into cells.^68^ In a study by Nagarajan et al., paraoxonase 2 (PON2) promoted tumor cell growth and metastasis by activating SLC2A1/GLUT1‐mediated glucose transport and inhibiting the AMPK/FOXO3A/PUMA pathway.^69^ According to another study, overexpression of GLUT‐1 upregulated the expression of MMP‐2 and promoted pancreatic cancer cell invasiveness.^70^ The prognostic value of GLUT1 has also been explored by Sharen et al., who found that a high level of GLUT1 expression was associated with a tumor size greater than 2 cm and with lymph node metastasis in patients with pancreatic cancer, and these features resulted in shorter overall survival.^71^

In addition to its involvement in pancreatic cancer angiogenesis, the sodium bicarbonate transporter SLC4A4 is also related to invasiveness.^63^ Zhang et al. reported that overexpression of human circular RNA 001587 inhibits the migration and invasion of pancreatic cancer cells, changes that are counteracted by upregulating the expression of SLC4A4.^63^

As mentioned above, SLC7A11 encodes a subunit of the amino acid transport system X_c_ that mediates cystine uptake and glutamate biosynthesis.^26^ In addition to its function in proliferation, Zhu et al. also found that overexpression of SLC7A11 promotes tumor cell invasion and metastasis, changes that are suppressed by miR‐139‐5p via the PI3K/Akt signaling pathway (Figure 2).^27^ Furthermore, the authors verified this result with clinical data showing that low expression of miR‐139‐5p and high expression of SLC7A11 are associated with a poor prognosis, indicating that miR‐139‐5p may represent a new biomarker of pancreatic cancer.^27^

SLC9A1, also known as Na^+^/H^+^ exchanger isoform 1 (NHE1), is a membrane transporter regulating pH in tumor cells, and its main function is to maintain the proton gradient.^72^ A study by Cardone et al. showed that NHE1 interacts with epidermal growth factor receptor (EGFR) to promote the growth and invasion of pancreatic cancer tumor cells via the digestion of the extracellular matrix.^73^

A study by Olszewski et al. also showed that neurotensin receptor signaling promotes pancreatic cancer cell metastasis by activating SLC9A1.^74^ Malinda et al. observed upregulated expression of SLC9A1 and SLC4A7 during the aberrant transforming growth factor β (TGFβ)‐induced EMT in pancreatic cancer cell lines.^75^

A recent study by Liu et al. substantiated the role of SLC39A4 in pancreatic cancer progression.^76^ Their results showed that overexpression of SLC39A4 promoted migration and invasion in pancreatic cancer by downregulating the expression of ZO‐1 and Claudin‐1 through upregulation of the expression of transcriptional repressor ZEB1.^76^ Moreover, SLC39A6 encodes the protein LIV‐1. A study by Unno et al. indicated that LIV‐1 might be involved in tumor invasion and metastasis through the EMT.^35^

### 2.5 Cellular metabolism

The uncontrolled cell proliferation in neoplastic diseases increases their demand for nutrients to fuel cell growth and division, and they meet these needs by reprogramming metabolism.^16^ Tumor cells must upregulate the expression of the transporters responsible for the uptake or secretion of nutrients such as glucose, lactate, and amino acids to alter metabolism.^77^ Otto Warburg first observed the abnormal energy metabolism or “aerobic glycolysis” of cancer cells; namely, tumor cells reprogram their metabolism by oxidizing glucose and producing lactate as waste mainly through glycolysis under aerobic conditions.^16^, ^77^ Tumor cells must upregulate the expression of the glucose transporters SLC2A1 (GLUT1) and SLC5A1 (SGTL1) to increase glucose uptake.

Many researchers have reported the overexpression of the SLC2A1 gene in patients with pancreatic cancer compared to normal controls.^78^, ^79^ Brandi et al. reported that the antioxidant mitochondrial uncoupling protein 2 (UCP2) promotes the proliferation of pancreatic cancer cells by upregulating the expression of SLC2A1 to limit metabolism to glycolysis.^80^ Nagarajan et al. also found that human paraoxonase 2 (PON2) promotes pancreatic cancer cell growth and metastasis through SLC2A1‐mediated glucose transport.^69^ The prognostic value of this gene has been studied, and the results indicate that the expression of SLC2A1 is negatively correlated with the prognosis of patients with pancreatic cancer.^68^, ^71^, ^81^, ^82^

Another glucose transporter, SLC5A1, encodes sodium‐dependent glucose transporter 1 (SGLT1). Gao et al. observed SLC5A1 overexpression in pancreatic cancer cells, and knockdown of this gene suppresses the growth and progression of pancreatic cancer by reducing glucose uptake through the AMPK/mTOR signaling pathway.^83^ The prognostic value of SLC5A1 has also been illustrated by the significant association between the high expression of SLC5A1 in pancreatic cancer and the disease‐free survival (DFS) of patients.^84^

Tumor cells also need to increase the uptake of amino acids to fuel cell growth. The induction of SLC1A5 expression by hypoxia promotes the transport of mitochondrial glutamine to facilitate metabolism reprogramming in pancreatic cancer.^17^ A study by Hayashi et al. showed an important role for SLC7A5 in tumor cell growth in pancreatic cancer.^25^ The prognostic significance of this gene has been illustrated by the association of high SLC7A5 expression with shorter overall survival in patients with pancreatic cancer.^85^, ^86^

In addition to glucose and amino acids, nucleotides are also quite important for tumor cells. Vesicular ATP release through exocytosis is the main process inducing purinergic chemical transmission, which has also been shown to play a crucial role in regulating the function of pancreatic exocrine cells, from which PDACs most likely originate.^87^, ^88^ The purinergic signaling that transmits signals from nucleotides, particularly ATP, ADP, and adenosine, affects several steps of tumor biology ranging from the tumor microenvironment to proliferation, migration, EMT and metastasis, energy metabolism, and immune evasion.^89^

The vesicular nucleotide transporter (VNUT) encoded by the SLC17A9 gene mediates vesicular ATP accumulation and release in secretory granules in different tissues with abundant vesicular ATP transporters, such as the brain, adrenal glands, pancreas, and other tissues.^87^, ^90^, ^91^ Novak et al. summarized the purinergic signaling in pancreatic cancer and indicated that PDAC cell lines release ATP through VNUT in response to several stimuli.^92^ Furthermore, VNUT regulates the viability of pancreatic cancer cells by functioning as lysosomal ATP transporters, and extracellular nucleosides also promote anticancer immunity by inducing immunogenic cell death.^93^, ^94^

### 2.6 Chemosensitivity

The current cornerstones of chemotherapy in patients with pancreatic cancer are the uracil analog 5‐fluorouracil (5‐FU) and the cytidine analog gemcitabine (GEM). In the past few decades, with the emergence of new chemotherapy drugs, such as tegafur/gimeracil/oteracil potassium capsule (S‐1), albumin‐bound paclitaxel (Abraxane), oxaliplatin and irinotecan, more options have been developed for combined chemotherapy for the treatment of pancreatic cancer. However, the prognosis remains poor because of multidrug resistance (MDR), with a 5‐year relative survival rate of less than 8%.^95^ MDR is the major obstacle that affects the efficacy of systematic treatment in patients with pancreatic cancer. Because the SLC family transports a wide variety of substrates, including anticancer drugs, the mechanisms by which SLC proteins modulate MDR are mainly due to the decrease in drug uptake into tumor cells mediated by the low expression of certain SLCs.^96^, ^97^

In addition to its function in sustained proliferative signaling, SLC1A5 also plays a role in chemotherapy sensitivity. A study by Yoo et al. showed that overexpression of an SLC1A5 variant conferred resistance to gemcitabine in pancreatic cancer cells.^17^

SLC7A11 functions in proliferation and invasion, but Yang et al found that overexpression of the antisense transcript of SLC7A11 (SLC7A11‐AS1) in pancreatic cancer cells promotes gemcitabine resistance by reducing ROS levels through the inhibition of the ubiquitination and degradation of nuclear factor erythroid‐2‐related factor 2 (NRF2).^98^ Similarly, Lo et al. also found that upregulation of SLC7A11 (X_c_^−^) is associated with gemcitabine resistance.^99^

SLC22 subfamily members such as organic cation transporter 1 (OCT1, SLC22A1), OCT2 (SLC22A2), and OCT3 (SLC22A3) have been reported to transport platinum agents in vitro, but the results remain controversial.^100^ Mohelnikova‐Duchonova et al. reported the upregulated expression of SLC22A3 in pancreatic cancer tissues compared with nonneoplastic tissues, and this change predicted a favorable overall survival in patients treated with 5‐FU or gemcitabine‐based chemotherapy (*p *= .004).^101^

SLC28, a concentrative nucleoside transporter (CNT) subfamily member, was also reported to mediate the transport of gemcitabine and 5‐FU in pancreatic cancer.^102^, ^103^, ^104^ Mackey et al. identified important roles for SLC28A1 and SLC28A2 in gemcitabine transfer in vitro and showed that a deficiency in SLCs might contribute to gemcitabine resistance.^105^ García‐Manteiga et al. concluded that most cell lines take up gemcitabine via SLC29A1 instead of SLC28A1, which is expressed in human pancreatic cancer at high levels, and that the expression of SLC28A1 and SLC29A1 may increase the sensitivity of pancreatic cancer cells to gemcitabine.^102^

SLC29A1, also known as equilibrative nucleoside transporter 1 (ENT1), mediates both the influx and efflux of nucleosides across the membrane. According to previous studies, pancreatic cancer cell lines take up gemcitabine via SLC29A1.^102^ A large number of studies have documented that high SLC29A1 expression is associated with a favorable prognosis for patients with pancreatic cancer undergoing gemcitabine chemotherapy.^106^, ^107^, ^108^ Furthermore, the prospective randomized studies ESPA‐3 and RTOG9704 are assessing this gene, and results have shown a significantly longer median survival for patients undergoing gemcitabine chemotherapy with high SLC29A1 protein expression compared with patients with low or negative SLC29A1 protein expression.^109^, ^110^ However, the number of studies is small, and inconsistent results have been obtained.^111^ Kawada et al. did not observe an association between the expression level of SLC29A1 and the prognosis of patients with pancreatic cancer treated with neoadjuvant gemcitabine chemotherapy, potentially due to the limitation that the SLC29A1 evaluation occurred after neoadjuvant chemoradiation.^111^ A randomized multicenter phase II study also showed that SLC29A1 did not predict gemcitabine sensitivity in patients with metastatic pancreatic cancer.^112^ A multicenter phase III study of AIO‐PK0104 from Germany comparing two adjuvant chemotherapy regimens in patients with advanced pancreatic cancer reported similar results.^113^ In general, the role of SLC29A1 in gemcitabine chemotherapy resistance remains controversial and requires further study.

**FIGURE 4:**
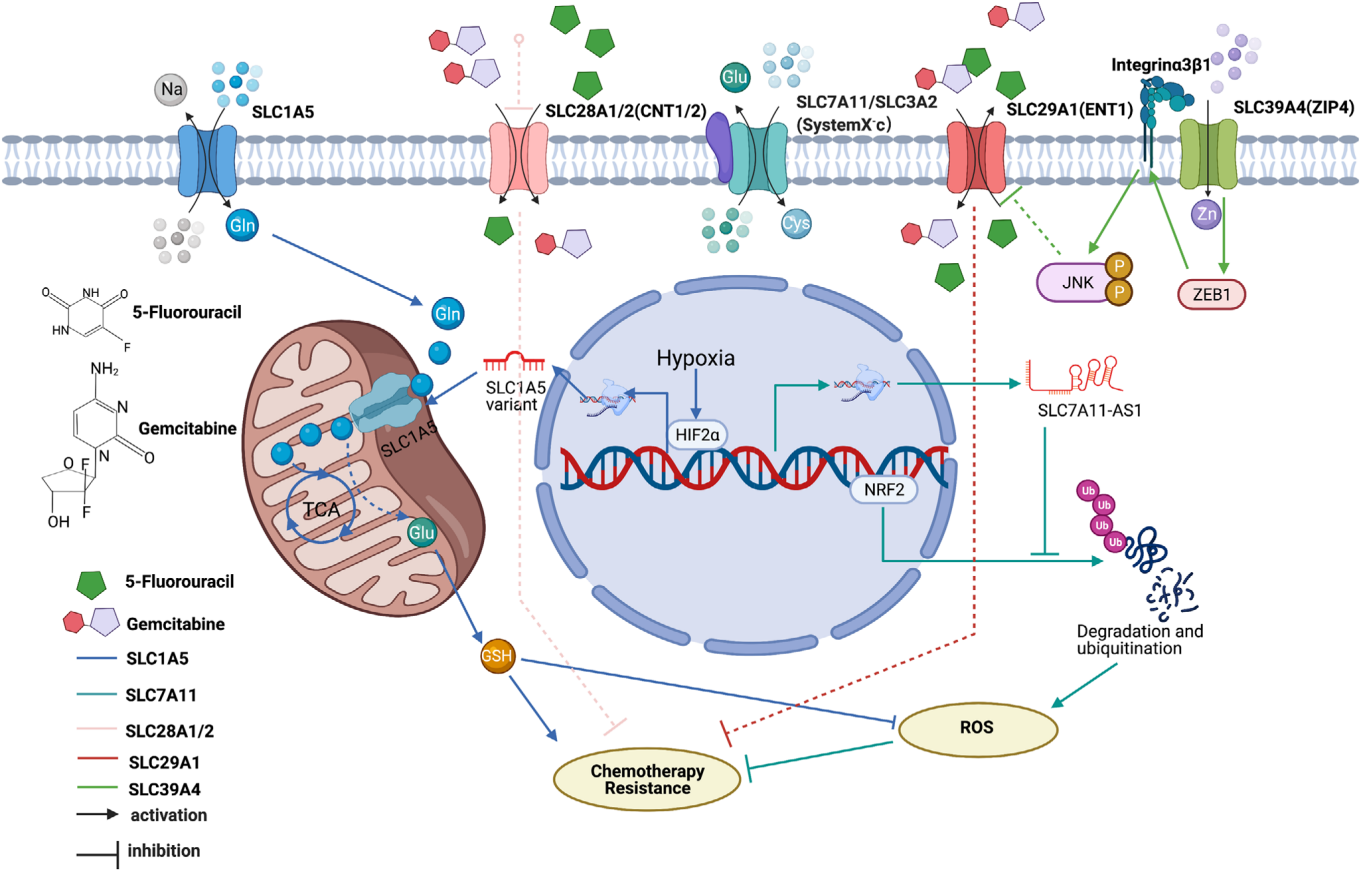
Solute carrier (SLC) expression is associated with chemotherapy resistance. (A) The expression of an SLC1A5 variant that encodes the mitochondrial glutamine transporter is controlled by hypoxia through HIF2α. Overexpression of the SLC1A5 variant confers resistance to gemcitabine in pancreatic cancer cells.^17^ (B) The expression of the antisense transcript of SLC7A11 (SLC7A11‐AS1) in pancreatic cancer cells promotes gemcitabine resistance by reducing ROS levels through the inhibition of the ubiquitination and degradation of NRF2.^98^ (C) Deficiency in SLC28A1 and SLC28A2, which mediate the transport of gemcitabine and 5‐FU in pancreatic cancer, contributes to chemotherapy resistance.^105^ (D) Pancreatic cancer cells take up gemcitabine and 5‐FU via SLC29A1.^102^, ^114^ ZIP4 upregulates the expression of ZEB1 to suppress the expression of SLC29A1, which reduces gemcitabine uptake in pancreatic cancer cells^117^

Additionally, 5‐FU sensitivity has also been reported to be associated with SLC29A1 expression.^114^ This sensitivity is significantly increased when SLC29A1 is inhibited by nitrobenzylmercaptoprine ribonucleoside (NBMPR).^114^ Nevertheless, these results are not consistent with those of the prospective studies RTOG9704 and ESPAC‐3.^109^, ^110^ Limited data are available on other members of the SLC29 subfamily. Nishio et al. found that a change in localization of SLC29A2 disrupted gemcitabine uptake, resulting in chemotherapy resistance in pancreatic cancer.^115^ High expression of SLC29A3 is significantly associated with favorable overall survival in patients with pancreatic cancer, but the underlying mechanism remains unknown.^101^ Unlike SLC29A1 and SLC29A2, Endo et al. reported that SLC29A3 and SLC29A4 are not prominent nucleoside transporters and that a significant improvement in chemotherapy sensitivity is not observed in cell lines expressing these two genes.^116^ ZIP4 upregulates the expression of ZEB1 to suppress the expression of SLC29A1, a gemcitabine transporter, which reduces gemcitabine uptake in pancreatic cancer cells (Figure 4).^117^

## 3 FUTURE PERSPECTIVES

With the gradual discovery of the functions of some SLCs over the past few decades, SLCs have increasingly become targets of anticancer drugs and a future hotspot of this field.^118^, ^119^ The advantages of SLCs as anticancer drug targets might be their easily accessibility without crossing any cellular barriers and the ability to reduce the cytotoxicity caused by nonselective drugs.^11^ SLCs encode over 40% of transporter proteins, and more than 80 SLCs have been reported to be associated with genetic disorders, but only a small number of these proteins have been approved or are currently undergoing testing.^120^ According to review by Zhang et al., drugs targeting the SLC5, 6, 12, 18, 22, 25, and 29 subfamilies have been approved as treatments for hematological, nervous system, motor, and other diseases.^121^ An additional subfamily of nine SLCs, including SLC2, 5, 7, 9, 10, 22, and 40, is being tested in clinical trials.^121^ However, the potential of SLCs in precision treatment for pancreatic cancer still remains uncertain but has great therapeutic potential, and prospective clinical trials are needed to support the results of basic research studies.

SLCs regulate metabolic homeostasis, but researchers have not yet determined whether they alter the immune microenvironment in pancreatic cancer. According to the immunosurveillance theory, tumor cells have the ability to avoid detection and evade eradication by the immune system. The depletion of L‐Arg in T cells inhibits mTOR through SLC38A9, which in turn suppresses the growth and proliferation of T cells.^122^, ^123^, ^124^ Kynurenine, which is transported by SLC7A5, SLC7A8, and SLC36A4, can lead to Trp degradation in effector T cells, subsequently impairing the immune function and inducing PD‐1 expression.^125^, ^126^ Furthermore, tumor cells have also been reported to outcompete T cells for methionine by expressing a high level of the transporter SLC34A2.^127^ However, no studies have examined the role of SLCs in the immune evasion of pancreatic cancer, necessitating in‐depth studies to decipher this problem. We believe this topic will be a hot area in the future.

## 4 CONCLUSIONS

In summary, SLCs play various roles in pancreatic cancer, including proliferative signaling, cell death, angiogenesis, tumor invasion and metastasis, cellular metabolism, and chemotherapy sensitivity, providing possible therapeutic strategies for cancer treatment. Although an increasing number of studies have revealed the important functions of SLC family members in tumor biology, they have received relatively little attention to date. The functions and substrates of many SLCs remain unknown. In addition, the data are inconsistent and the conclusions are ambiguous. As a result, research on SLCs in pancreatic cancer is still in its infancy. Furthermore, membrane proteins are difficult to crystallize, which hinders the elucidation of the physiological function, and three‐dimensional structures need to identify substrate binding sites and important domains. Despite these obstacles, the therapeutic potential of SLCs in pancreatic cancer is substantial. We propose that with the development of molecular biology, new SLCs and new functions will be discovered and translation from basic advances into clinical treatment will become a reality.

## CONFLICT OF INTEREST

The authors declare that there is no conflict of interest.

## AUTHOR CONTRIBUTIONS

Zijian Wu, Jin Xu, and Chen Liang wrote the manuscript and created the illustrations. Qingcai Meng, Jie Hua, Wei Wang, Bo Zhang, and Jiang Liu contributed to the acquisition of literature. Si Shi and Xianjun Yu contributed to the conception and design, revised the article, provided final approval of the version to be published, and agreed to be accountable for all aspects of the work.
